# Scalable Optical Convolutional Neural Networks Based on Free-Space Optics Using Lens Arrays and a Spatial Light Modulator

**DOI:** 10.3390/jimaging9110241

**Published:** 2023-11-06

**Authors:** Young-Gu Ju

**Affiliations:** Department of Physics Education, Kyungpook National University, 80 Daehakro, Bukgu, Daegu 41566, Republic of Korea; ygju@knu.ac.kr; Tel.: +82-(53)-9505894

**Keywords:** optical neural network, convolutional neural network, free-space optics, optical computer, smart pixels

## Abstract

A scalable optical convolutional neural network (SOCNN) based on free-space optics and Koehler illumination was proposed to address the limitations of the previous 4f correlator system. Unlike Abbe illumination, Koehler illumination provides more uniform illumination and reduces crosstalk. The SOCNN allows for scaling of the input array and the use of incoherent light sources. Hence, the problems associated with 4f correlator systems can be avoided. We analyzed the limitations in scaling the kernel size and parallel throughput and found that the SOCNN can offer a multilayer convolutional neural network with massive optical parallelism.

## 1. Introduction

In recent times, the advent of artificial neural networks with deep learning algorithms has led to considerable advances in applications such as image and speech recognition and natural language processing [1,2]. The convolutional neural network (CNN) is a type of deep learning algorithm that is particularly effective in image and video analysis [3]. CNNs are specifically designed to automatically detect and extract features such as edges, corners, and textures from images; these features can be used to classify the images into different categories. These applications involve processing an input image by applying convolutional operations using kernels of different sizes. The results of these convolutions are then pooled, passed through a nonlinear activation function, and sent to the next layer of convolutional operations. Although CNNs are excellent at solving classification and recognition problems, they require a massive amount of computation, especially when dealing with large images and kernels. When an input image with n × n pixels is convolved with a kernel of size k × k, the amount of computation is proportional to (n^2^ × k^2^). The computational requirement grows further with an increasing number of layers, resulting in high latency and large power consumption in the case of forward inference in the pretrained network. Although the use of graphics processing units can alleviate the issue of latency, real-time inference may still remain a challenge [4].

Currently, researchers are exploring the use of free-space optics to implement CNNs in an optical form owing to the high parallelism and energy efficiency of these optics [5,6,7,8]. Optical convolutional neural networks (OCNNs) based on free-space optics traditionally use the well-known 4f correlator system to exploit the Fourier transform property [9]. Although these types of OCNNs have some advantages, they cause several inherent problems because of the use of Fourier optics. The first issue is the limitation in the scalability of the input image array; a lens is used for Fourier transformation, and the lens has a finite space–bandwidth product (SBP) owing to its geometric aberration. The second issue is the latency caused by the time taken to generate the input array. In a Fourier-transform-based system, a laser and a spatial light modulator (SLM) are required to generate a coherent input. However, most of the currently available SLMs are slow and serially addressable, resulting in considerable latency [10,11,12,13,14]. This latency diminishes the advantages of massive parallelism in the optical neural network. In addition, it poses a challenge in building a cascaded system for a multilayer neural network in which the output of one layer acts as the input for the next layer. However, in the 4f correlator system, which relies on a single coherent source, reconfiguration of the SLM pixels is necessary whenever the output of the layer changes and the input of the next layer correspondingly changes. Therefore, the slow refresh rate of the SLM considerably affects the throughput of the optical neural network. Despite recent advancements in micro-electromechanical systems (MEMS) technology, most SLMs still operate in the tens of kilohertz range, which is considerably slower than electronic switches. Therefore, the slow speed of the currently available SLMs causes considerable latency within the 4f correlator system. The third problem is the difficulty of reconfiguring the kernels. The kernel pattern on the 4f system is the Fourier transform of the kernel pattern; obtaining the Fourier transform requires computation and can lead to significant delays in renewal.

To address these issues, we propose a scalable optical convolutional neural network (SOCNN) based on free-space optics and Koehler illumination, which uses lens arrays and a SLM. The SOCNNs proposed herein are a variation of the optical neural network previously reported, known as a linear combination optical engine (LCOE) [15]. Furthermore, we incorporated the CNN architecture within the context of LCOE. The goal of the LCOE was full interconnection; in contrast, the goal of the SOCNNs was partial connection with an unlimited input array size.

## 2. Materials and Methods

**Figure 1:**
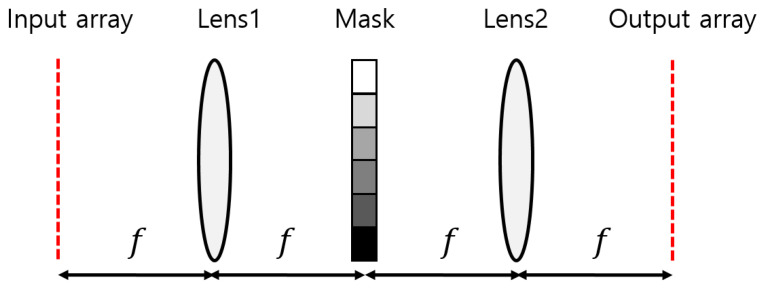
Example of a 4f correlator system that uses Fourier transform to implement an existing optical convolutional neural network (OCNN). The mask represents the Fourier transform of the kernel used in the CNN. *f* represents the focal length of the Lens1 and Lens2.

In a typical 4f correlator system, a mask is located at the focal plane of two lenses, as shown in Figure 1. Lens1 performs the Fourier transform, while Lens2 performs the inverse Fourier transform. The mask represents the complex-valued Fourier transform of a kernel, which is multiplied by the Fourier transform of an input pattern. Thus, the output plane displays the convolution of the input array and kernel.

The SBP of the 4f imaging system is approximately ${\left(\frac{D^{2}}{\lambda f}\right)}^{2}$, where *D*, *λ*, and *f* are the diameter of the lens, wavelength of the light source, and focal length of the lens, respectively [5]. The SBP can be expressed as ${\left(\frac{D}{\lambda \;f/\#}\right)}^{2}$ using the f-number (*f/#*) of the lens. If the fixed wavelength and *f/#* are used, the SBP can increase infinitely with *D*. However, this SBP arises from the diffraction limit of the lens used in the system. As *D* increases, it becomes difficult for the system to reach the diffraction limit. A larger system requires less geometric aberration, more elements, and tighter alignment tolerance to reach the diffraction limit. The SBP of the system is about ${\left(\frac{D}{2\;f\;\delta }\right)}^{2}={\left(\frac{1}{2\;f/\#\;\delta }\right)}^{2}$, where *δ* is the angular aberration of the lens. When a triplet lens has a f-number of 2, the angular aberration is approximately 3 mrad, and the SBP is about 83 × 83. Since a lens system can worsen alignment problems during assembly with an increase in the number of elements, the practical scaling limit of the 4f system can be about 250 × 250 if the angular aberration is 1 mrad.

**Figure 2:**
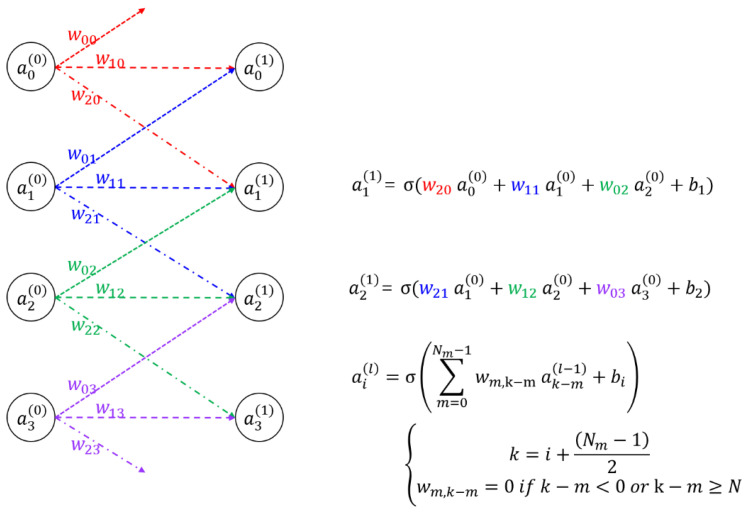
Example of a simple CNN with corresponding mathematical formula; $a_{i}^{\left(l\right)}$ represents the *i*-th input or output node in the *l*-th layer; $w_{ij}$ indicates the weight connecting the *j*-th input node and the *i*-th output node; $b_{i}$ is the *i*-th bias; *N* is the size of the input array; *N_m_* is the number of weights connected to an input/output or the size of a kernel; and σ is a sigmoid function.

To understand the architecture of the proposed SOCNN, it is essential to grasp the concept of CNN. An example of a CNN is shown in Figure 2; it shows four input nodes, four output nodes, and their synaptic connections along with mathematical representations. The CNN operates by receiving input signals through the input nodes, which are then transmitted through synaptic connections to the output nodes for processing; thus, the output is obtained. The strengths of the synaptic connections are modeled using mathematical representations that assign weights to each connection. In contrast to the full-connection optical neural network such as the LCOE, in the CNN, each input or output node has local or partial connections whose weights are called kernels.

**Figure 3:**
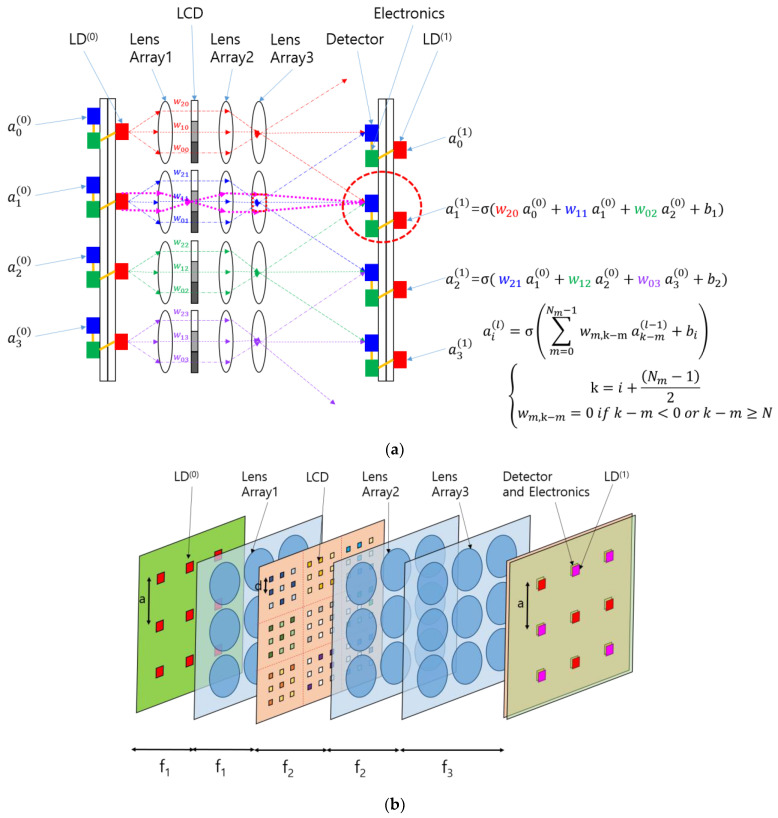

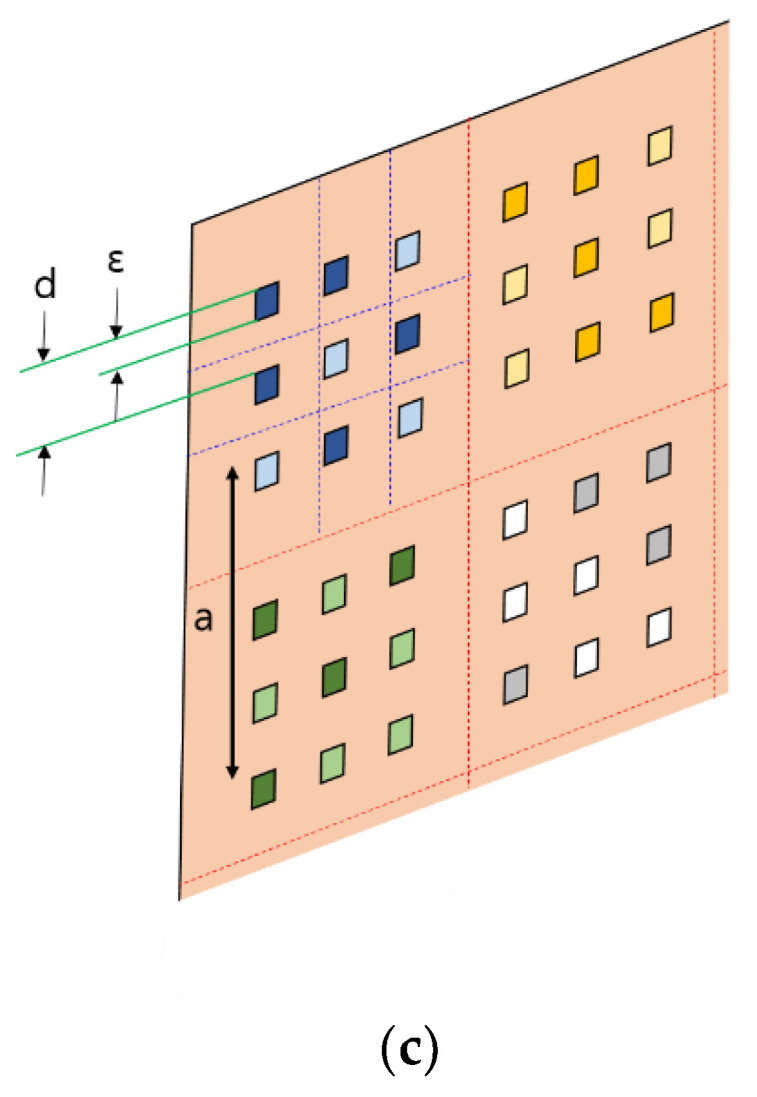
Scalable optical convolutional neural network (SOCNN) based on Koehler illumination and free-space optics using lens arrays and a spatial light modulator: (**a**) schematics and the corresponding mathematical formula; (**b**) three-dimensional view of a system with 3 × 3 inputs and outputs; and (**c**) the structural parameters of the SLM (LCD) pixels and their subarrays. The small, colored squares represent pixels.

The concept of the SOCNN proposed herein is illustrated in Figure 3a. The CNN shown in Figure 2 was transformed into a hardware schematic containing laser diodes (LDs), lenses, a liquid crystal display (LCD), detectors, and electronics. The input node was replaced with an LD that sent three rays to lens array 1. The LD used in this architecture can be a multimode laser diode or a light-emitting diode (LED) because, unlike the traditional 4f correlator system, this system accommodates incoherent light sources.

While we used an LCD as an example of an SLM in the proposed SOCNN, it is important to note that other types of SLMs can also be employed. However, SLMs based on micro-mirror or MEMS technology have certain drawbacks in system design [10,11,12,13,14]. They require input and output optical paths to be on the same side, which necessitates either sharing the optical path or using oblique light paths on the micro-mirror device. This complexity can be problematic, particularly in cascading systems such as multilayer optical neural networks, as it also requires more space. In contrast, LCDs offer a reasonable choice for the SOCNN as they are transmission-type, cost-effective, high-density pixels with mature and readily available technology.

Lens array 1 collimates the rays and sends them to the LCD, where each pixel transmits the corresponding ray according to a pretrained kernel in the CNN. The rays from the LCD pass through lens array 2, which focuses the rays and generates different ray angles depending on the distance of the LCD pixel from the optical axis of the individual lenses in the array.

A detector collects or adds the optical power of the rays arriving at different angles from different neighboring LDs or inputs with preset weights. In this scheme, the summed light is mathematically a convolution of the inputs and the kernel specified using the weights. These SOCNNs can perform the calculations in parallel and, most importantly, in one step with the speed of light if the weights and inputs are preset. This type of calculation is called “inference” in the neural network community. Although the SOCNNs based on an LCD are reconfigurable, they are more suitable for inference applications owing to the low switching speed.

A detailed examination of the optical system, as shown in Figure 3a, indicates that lens 2 and lens 3 form a relay imaging system in which the LCD is typically positioned at the focal plane of lens 2, while the detector is placed at the focal plane of lens 3. This arrangement ensures that the LCD and detector planes are conjugated—in other words, each pixel on the LCD forms an image on the detector plane. By establishing this conjugate condition, the illumination area of each ray is more clearly defined, and the crosstalk between channels is reduced.

Additionally, if an LD is placed at the focal plane of lens 1, lens 1 and lens 2 form a relay system, and an image of the LD is formed at lens 3. Overall, the lens configuration shown in Figure 3a constitutes a Koehler illumination system [16,17]. Figure 3a shows dotted magenta lines that represent the chief rays from the perspective of the condenser system and the marginal rays from the perspective of the projection system. The red-dotted rectangular block located at lens 3 represents the image of the light source. Lens 2 and lens 3 work together to form a projection lens in the Koehler illumination system. In contrast, the rays originating from the LD point of emission spread out over the detector plane, providing uniform illumination. Koehler-illumination-based optical computers have several advantages over previously reported architectures based on Abbe illumination in terms of uniformity of illumination and control of the beam divergence of light sources [15].

The key difference between this SOCNN and the previous LCOE is that each input of the SOCNN has a relatively small number of connections to the output array, whereas the LCOE has a full interconnection. The feature of partial connection greatly relieves the constraint on the size of the input array. In fact, unlike the traditional 4f correlator system, this SCONN does not have any theoretical limit on the input array size. Only the size of the kernel array is limited, since the SLM pixels used for the kernel are imaged through lenses that impose a constraint on SBP; this topic will be explained in detail in Section 3.

In the example shown in Figure 3a, the number of LCD pixels belonging to each input node is equal to the number of output nodes to which the input is connected. The LCD pixels belonging to each input node can be called a subarray of the SLM. The size of the subarray is the same as that of the receptive field from the viewpoint of the output node. Although the SOCNN depicted in Figure 3a appears to be one-dimensional, it can be extended to a two-dimensional input and output array. The two-dimensional mathematical formula is provided in Appendix A. In the case shown in Figure 3b, the subarray comprises a 3 × 3 array where the spacing between the pixels is *d*. Given that the spacing between the detectors is *a*, the magnification of the projection system should match the size of the SLM subarray. The magnification of the projection system in the SOCNN is written as *f*_3_/*f*_2_ using the notations shown in Figure 3b.

If an 8 × 8 pixel area on the LCD is assigned to a single kernel, it can be connected to 64 output nodes. For instance, an LCD with 3840 × 2160 resolution can accommodate up to 480 × 270 input nodes, which translates to 129,600 inputs. Considering the parallelism of the SOCNN, its performance is equal to the number of pixels in the LCD. If the system has N × N inputs and an M × M kernel, it can perform N^2^ × M^2^ multiplications and N^2^ × (M^2^ − 1) additions in a single step. If the SOCNN takes full advantage of the LCD resolution, (N × M)^2^ equals the total number of pixels in the LCD, and this number is immensely large in modern devices. This LCD can be replaced with other types of SLM arrays for achieving high speeds if a fast refresh rate of weights is required. Furthermore, since the transmission of SLM pixels in the SOCNN is proportional to the weight of the kernel, extra calculations for Fourier transform are not required, unlike in the 4f correlator system—this is another advantage of SOCNNs for use as reconfigurable OCNNs in the future.

After the optical process, the detector converts the light into current, and the remaining steps such as signal amplification, bias addition, and application of nonlinear functions (e.g., sigmoid, rectified linear units, local response normalization, and max pooling) are performed electronically. These nonlinear functions are better handled with electronics than with optics because of their inherent properties. However, when electronics are used, interconnections between far-neighboring electronics should be minimized to avoid traffic congestion. As long as the electronics employed are local and distributed, the optical parallelism of the system remains unaffected. The electronic part, including the detectors, is similar to the concept of smart pixels [18].

The proposed system is a cascading system, and it can be extended in the direction of beam propagation. The signal from the output node is directly connected to the corresponding input of the next layer, allowing a detector, its corresponding electronics, and an LD in the next layer to form a synaptic node in an artificial neural network. If the system has L layers, N^2^ × M^2^ × L calculations can be performed in parallel in a single step; this ability greatly increases the SOCNN throughput for continuous input flow.

In fact, the addition of incoherent light using a detector and an LCD cannot represent the negative weight of a kernel in a CNN. If coherent light and interference effects are used, the system can represent subtraction between inputs. However, the use of coherent light may complicate the system and increase noise. The previous OCNN, based on a 4f correlator system, used a coherent light source and an SLM to generate an input array. As mentioned in the introduction, this coherent source entails many problems such as latency, noncascadability, and noise. Handling negative weights with incoherent light sources in this study can be solved by using the “difference mode”, as described in previous references [15,19].

**Figure 4:**
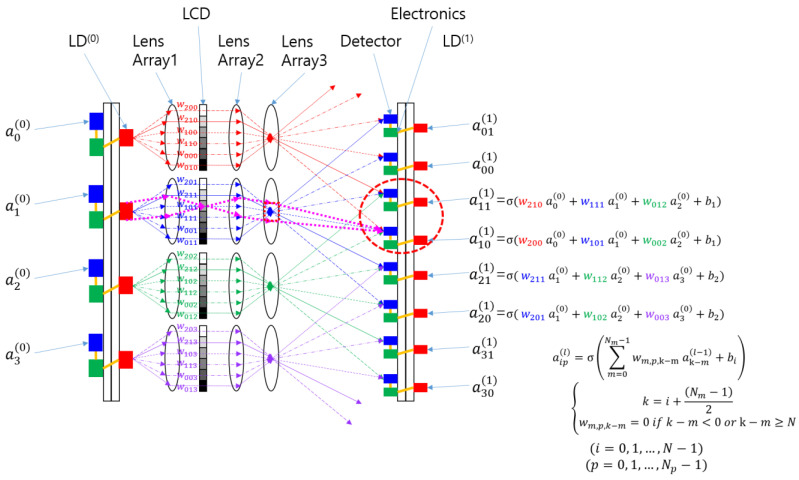
Difference mode configuration of the SOCNN; this mode can also be used for calculating multiple kernels for a single input array; and a generalized mathematical formula is given, where N_p_ represents the number of detectors corresponding to one of lens 3.

To implement the difference mode in the SOCNN, two detectors are required for each output node, or lens 3 with two separate channels indicated by a red dotted circle is required, as shown in Figure 4. The two optical channels separate the inputs with positive weights from those with negative weights. Each channel adds input values multiplied by their respective weights using optical means. Subsequently, subtraction between the two channels is performed electronically through the communication between neighboring electronics. Note that the weight in the negative channel should be zero when the corresponding positive weight is used, and vice versa. For example, if $w_{20}$ and $w_{02}$ are positive, and $w_{11}$ is negative, as shown in Figure 3a, then Equations (1) and (2) are used to represent the positive and negative weight calculations, respectively, as shown in Figure 4.

(1) $$w_{200}=w_{20}\;,w_{002}=w_{02}\;,{\;w}_{101}=0$$

(2) $$w_{210}=0=w_{012}\;,{\;w}_{111}={-w}_{11}$$

This subtraction scheme simplifies the structure but requires an additional channel.

To implement other functions, such as multiple kernels for the same input array, more than two detectors can be used for one of lens 3. The number of detectors corresponding to each lens is denoted as *N_p_* in Figure 4. For easy reference, the detectors associated with each lens 3 can be referred to as a “page of detectors”. Since Figure 4 represents a one-dimensional configuration, *N_m_* and *N_p_* can be generalized into M × M and P × P, respectively, in a two-dimensional configuration. In this case, the array size of the subarray corresponding to one input node becomes (M × P)^2^. Suppose that the spacing between pixels and that between the subarrays are denoted as *d* and *a*, respectively. The side length of the subarray is MP*d*. Since P detectors are arranged along length *a*, the detector spacing *d*_2_ is equal to M*d*. This means that the magnification of the projection system consisting of lens 2 and lens 3 should be M. Thus, a page of detectors can be implemented for difference mode or multiple kernels.

## 3. Results

Although theoretically, the SOCNN has no limit on the input array size, scaling the size of the kernel is limited due to lens 3. The limit of the scaling can be analyzed using the method described in a previous report [15]. This analysis involves calculating the image spreading of the SLM pixel through the projection system in terms of geometric imaging, diffraction, and geometric aberration. The overlap between the images of neighboring pixels and the required alignment tolerance can be estimated from the calculated image size. The analysis begins by examining an example system with a SOCNN architecture, and this is followed by an exploration of the factors that limit the system’s scale-up and how diffraction and geometric aberration affect this scaling.

To simplify the analysis, we investigated the architecture shown in Figure 3a instead of that shown in Figure 4. The scaling analysis can be easily generalized to a page detector scheme. The proposed SOCNN is a system with two-dimensional (2D) input and output and a four-dimensional (4D) kernel such that the numbers of pixels are N^2^, N^2^ × M^2^, and N^2^ for the input array, SLM array, and output array, respectively, where N and M are the number of rows in the square input array and the kernel array, respectively.

If all three array components have the same physical size, the densest part is the SLM array. Therefore, it is better to initially design an array of SLM or LCD pixels for an example system. For the example system, we assumed a 5 × 5 array for the kernel. We assumed that the SLM had 5 μm square pixels, which were placed with a period of 20 μm in a rectangular array. According to the notations in Figure 3c, ε and *d* are 5 and 20 μm, respectively.

The 5 × 5 SLM subarray accepts light from a single light source through a single lens. The diameter of each lens in the lens array and the side length of the SLM subarray are both 100 μm, and they are denoted by *a* in Figure 3b,c. The distance *a* is also the pitch of lens array 1, lens array 2, and the detector. Lens 2 is supposed to have an f/# of 2. Because the SLM pixel was at the front focus of lens 2 and the detector was at the back focus of lens 3, the image of the SLM pixel was formed at the detector plane. Since detector pitch *a* = 5*d*, the magnification of the projection system should be 5. In general, if the subarray size of the kernel is M × M, *a* is equal to M*d*, and the magnification should be M.

The magnification of this projection relay system was a ratio of the focal length (f_3_) of lens 3 to the focal length (f_2_) of lens 2, i.e., f_3_/f_2_ = M. Therefore, the geometric image size of a pixel without aberrations and diffraction was Mε. Because the pitch of the SLM pixel array was magnified by the pitch of the detector array, the duty cycle of the image of the SLM pixel was ε/*d*, or 25% of the detector pitch, which is the same as the SLM pixel pitch. Therefore, the duty cycle of the geometric image in the detector pitch remains constant regardless of M, which is the kernel size.

The real image of one SLM pixel was enlarged via diffraction and aberration in addition to the geometric image size. The beam diameter that determined the diffraction limit was the image size of the light source for the condenser system composed of lens 1 and lens 2 according to the Koehler illumination concept. However, because the image size of the light source could be as large as the diameter of lens 1 or lens 2, the beam diameter of the relay system comprising these lenses should be assumed to be the diameter of lens 2 (D = *a*). The spot diameter attributed to diffraction was 2 λ f_3_/D = 2 λ M f_2_/D = 2 λ M f_2_/#, which was approximately 10 μm for a wavelength of 0.5 μm. The duty cycle of the diffraction spread in the detector pitch can be obtained by dividing the size of the diffraction spot with the detector pitch M*d*. The duty cycle of the diffraction spread corresponds to (2 λ f_2_/#)/*d*, or 10%, in terms of either the SLM pixel pitch or the detector pitch. The general formula for the duty cycle of diffraction spread is independent of M. Hence, the duty cycle of the diffraction origin is kept constant for a fixed f_2_/# when scaling up.

The effect of geometric aberration on the image spread can be investigated by assuming that f_2_/# is fixed during scaling. Since f_3_/# = M f_2_/#, f_3_/# increases with the scaling factor M. The spherical aberration, coma, astigmatism, and field curvature are proportional to the third power, second power, first power, and first power of 1/(f/#), respectively [17,20]. In other words, the angular aberration δ_3_ of lens 3 decreases with scaling. However, because f_2_/# remains constant, the angular aberration due to lens 2 becomes dominant. If lens 2 has an f-number of 2 and comprises three elements, the angular aberration δ_2_ is about 3 mrad. The image spread due to the geometric aberration is f_3_ δ_2_ = M f_2_ δ_2_ = M D_2_ (f_2_/#) δ_2_ = M^2^ *d* (f_2_/#) δ_2_. Since the detector pitch is M *d*, the duty cycle of the image spread in the detector pitch due to geometric aberration is M (f_2_/#) δ_2_. This value increases with scaling. When M and f_2_/# are 5 and 2, respectively, the geometric aberration of lens 2 with 3 elements accounts for 3% of the duty cycle. If the maximum duty cycle of the image spread is 40%, the maximum M is about 66. In this case, the alignment tolerance is a duty cycle of 25% because the geometric image size and the diffraction spread are 25% and 10%, respectively. The duty cycle of 25% corresponds to 5 μm in the SLM plane—this value is usually feasible to achieve in terms of optomechanics.

As more elements are used for lens 3, the angular aberration can be reduced, and the maximum M can be increased. However, for a larger number of elements, tighter alignment tolerance and higher difficulty for the assembly of the lens unit are necessary. From the viewpoint of a full-connection optical neural network such as the LCOE, an M value of 66 may be small. However, from the viewpoint of OCNN, which aims for partial connection, an M value of 66 is very large.

In addition, if M is a relatively small number, as is usually the case for kernels in practice, the burden of optics and their alignment can be drastically reduced. For instance, if M = 5, a simple planoconvex lens can be used for lens 2. For a planoconvex lens with an f-number of 8, the angular aberration is only 3 mrad, which is the same as that of an f/2 lens with three elements. In this case, when using the abovementioned SLM pixels, lens 2 and lens 3 have focal lengths of 800 μm and 4.0 mm, respectively, with a diameter of 100 μm. Generally, a larger M value results in a substantial increase in the focal length of lens 3, since f_3_ = M^2^ (f_2_/#) d. However, for small M values, f_3_ is within a reasonable length range and may simplify the optics.

Furthermore, the tangent value of the half-field angle of lens 3 can be obtained by dividing a half-field size of M *a*/2 by f_3_. Since f_3_ = M f_2_, the tangent of the half-field angle is *a*/(2 f_2_) = 1/(2 f_2_/#); thus, it is independent of scaling factor M. When f_2_/# = 2, the half-field angle is about 14°, which is within a reasonable range. The half-field angle decreases as f_2_/# increases, implying less aberration and less of a burden for optics related to the field angle.

## 4. Discussions

The concepts used in the proposed SOCNN architecture are related to those of lenslet array processors (LAP) [19]. A pseudoconvolution LAP in direct configuration was reported in [19]. The primary distinction between the LAP and SOCNN is that SOCNN uses three layers of lens arrays with more emphasis on distributed electronics and neural network applications, while LAP uses only a single-layer lens array. From the viewpoint of illumination, the SOCNN is based on Koehler illumination, whereas the LAP is based on Abbe illumination [16,17]. Koehler illumination provides better uniformity in the detector area than Abbe illumination; this uniformity is especially beneficial when dealing with nonuniform sources such as the LED and multimode LDs. Unfortunately, a detailed description or design of the illumination scheme is not provided in [19]; such a description or design is critical to the convolution performance. The divergence of the light sources and their control are not specified for the input array in the pseudoconvolution LAP scheme, though they determine the coverage of convolution or the size of the kernel.

The parallel throughput of the SOCNN depends on the size of the SLM, as discussed in Section 2. The SLM array can be divided into subarrays, depending on the size of the kernel. For a given size of the SLM array, a smaller kernel size leads to a larger input array size. Additionally, the SLM can be divided to accommodate multiple kernels by copying the input array into multiple sections. In fact, each section of the SLM can handle different kernels and perform convolution in parallel [5]. Therefore, the number of calculations per instruction cycle is equal to the number of SLM pixels. If the SLM has a resolution of 3840 × 2160, the total number of connections is approximately 8.3 × 10^6^, which is also the number of multiply and accumulate (MAC) operations in one instruction cycle. If electronic processing is assumed to be the main source of delay, with a delay time of 10 ns, the proposed optical computer in this study can achieve a throughput of 8.3 × 10^14^ MAC/s. This throughput can be further increased by using multiple layers. Although multiple layers may cause delays in data processing, all layers perform calculations simultaneously, similar to the pipelining technique used in digital computers. As the number of layers increases, the total throughput can also increase. Therefore, the proposed optical computer can achieve a throughput of 8.3 × 10^15^ MAC/s when 10 layers are used.

To achieve massive parallel throughput, it is crucial to input 2D data in parallel at every instruction cycle. If the 2D input is generated by serially reconfiguring individual pixels of the SLM, the parallelism of the optical computer is considerably reduced, similar to the case of the 4f correlator system. The 4f correlator system suffers from serialization between layers when used in a cascading configuration because the input of the next layer can be generated with a single coherent source and SLM array. However, in the SOCNN architecture, this serialization problem occurs only in the first layer, not between the layers, as it can use incoherent, independent light sources for input.

A possible solution to address the problem of serialization involves initializing the initial layer with a detector array and generating the 2D input in real-time using optical imagery, as proposed in a prior research study [15]. For instance, imaging optics can capture images of a moving object on the detector array, which then acts as the first layer for an optical neural network. Thus, serialization or deserialization of input data is unnecessary throughout the entire system because all the inputs in the following layers are fed from local electronics. This approach is similar to that of the human eye and brain, where the eye forms an image of the object on the retina, which is the first layer of the neural network that is connected to the brain. Since the SOCNN is used for the front-end of the optical neural network, this approach appears very reasonable.

## 5. Conclusions

Traditionally, 4f correlator systems have been employed in optical computing to perform convolution by conducting Fourier transforms with the use of two lenses. However, these systems exhibit limitations when it comes to scaling up the input array due to geometric aberrations. Moreover, implementing them necessitates a single coherent light source in conjunction with an SLM to generate the input, which introduces complexity and latency, particularly in the implementation of multilayer OCNNs with cascading configurations. Furthermore, the use of a Fourier-transformed kernel within the mask between two lenses entails additional calculation time and can introduce latency in future systems requiring higher refresh rates.

To address these issues, the SOCNN architecture was proposed. This architecture takes advantage of the Koehler illumination and comprises three lens arrays that form images of SLM pixels on the detector plane. The Koehler illumination scheme offers advantages over the previous Abbe illumination-based LAP architecture by providing more uniform illumination and lower crosstalk between the detectors.

The key advantages of the SOCNN are the scalability of the input array and the use of an incoherent light source. These advantages help avoid many problems inherent to the use of coherent sources, as in the case of the 4f system. As a partial connection version of LCOE, the SOCNN inherits many advantages of LCOE, which is also based on free-space optics and Koehler illumination. Compared with the LCOE, the SOCNN has a smaller coverage of connection to the output; in the SOCNN, the use of the last lens limits only the kernel size, not the size of the input array. This is the major advantage of the SOCNN over the existing 4f systems in terms of scaling up and parallel throughput. Another advantage of the SOCNN is that the weights of the kernel are directly set using the proportional transmission of SLM pixels, unlike the 4f system, which requires Fourier transform and causes latency in the future reconfigurable system.

Although the SOCNN has an extensively scalable input array, there is a limit to scaling the kernel size because the kernel information spreads out through a lens. The scaling limit of the kernel array was analyzed by observing the effect of changes in geometric image size, diffraction, and geometric aberration on the final image size. As M, i.e., the number of rows in the kernel array, increases, the duty cycle of the geometric image size and diffraction spread in the detector pitch remain constant for a fixed f-number of lens 2. In contrast, the duty cycle of image spread due to geometric aberration is proportional to M. When M is about 66, the duty cycle due to geometric aberration is equal to 40%, and the alignment tolerance has a duty cycle of 25%, which corresponds to 5 μm in the SLM plane. Usually, convolution does not require such a large array size, and hence, M = 66 seems sufficiently large for practical applications.

To estimate the parallel throughput of the SOCNN architecture, an example system was considered. The number of calculations per instruction cycle is equal to the number of SLM pixels. Assuming that electronic processing requires 10 ns and the system has 10 convolution layers, SLMs with a resolution of 3840 × 2160 can achieve a parallel throughput of 8.3 × 10^15^ MAC/s. SLMs with this resolution can accommodate up to 760 × 432 input nodes for a 5 × 5 kernel array. An advantage of the SOCNN is its considerably smaller diameter of lens 3 compared to the LCOE. The reduced diameter of lens 3 results in a smaller focal length for a given f/#. For instance, if lens 3 is a simple planoconvex lens with an f/# of 8, then its diameter and focal length should be 100 and 800 μm, respectively, assuming a SLM pixel pitch of 20 μm. The smaller focal length of lens 3 reduces the distance between neural network layers, contributing to the compactness of the system. Due to the capability of the SOCNN to handle large input arrays and its relatively easy and compact implementation, it is well-suited for practical applications as an optical preprocessor when combined with image sensors and existing electronic neural network chips, even before full optical neural networks become a reality. This SOCNN architecture can accelerate real-time image and video analysis, particularly in applications such as autonomous vehicles.

In summary, a SOCNN based on free-space optics and Koehler illumination was proposed to overcome the challenges in the previous 4f correlator system. The results reported herein imply that the SOCNN can offer a multilayer CNN with massive optical parallelism.

## Data Availability

The datasets generated and/or analyzed during the current study are available from the corresponding author on reasonable request.

## Appendix A

The mathematical formula shown in Figure 3a can be generalized into a two-dimensional form as follows. It is assumed that the sizes of the input array and kernel are *N_x_* × *N_y_* and *N_m_* × *N_n_*, respectively. Here, $a_{ij}^{\left(l\right)}$ represents the value of the *i*-th row and the *j*-th column node in the input or output for the *l*-th layer.

$$a_{ij}^{\left(l\right)}=\sigma \left(\sum_{m=0}^{N_{m}-1}\sum_{n=0}^{N_{n}-1}w_{mn,(k-m)(l-n)}a_{\left(k-m)(l-n\right)}^{\left(l-1\right)}+b_{ij}\right)\;\left\{\begin{array}{c} \begin{array}{c} k=i+\frac{\left(N_{m}-1\right)}{2}\\ w_{mn,(k-m)(l-n)}=0\;if\;k-m<0\;or\;k-m\geq N_{x} \end{array}\\ \begin{array}{c} l=j+\frac{\left(N_{n}-1\right)}{2}\\ w_{mn,(k-m)(l-n)}=0\;if\;l-n<0\;or\;l-n\geq N_{y} \end{array} \end{array}\right.$$
